# Current Concepts of Neurodegenerative Mechanisms in Alzheimer's Disease

**DOI:** 10.1155/2018/3740461

**Published:** 2018-03-08

**Authors:** Kasthuri Bai Magalingam, Ammu Radhakrishnan, Ng Shee Ping, Nagaraja Haleagrahara

**Affiliations:** ^1^Department of Pathology, Faculty of Medicine, International Medical University, 57000 Kuala Lumpur, Malaysia; ^2^Department of Applied Sciences, Nilai University, 71800 Negeri Sembilan, Malaysia; ^3^College of Public Health, Medical and Veterinary Sciences, James Cook University, Townsville, QLD, Australia

## Abstract

Neurodegenerative diseases are hereditary or sporadic conditions that result in the progressive loss of the structure and function of neurons as well as neuronal death. Although a range of diseases lie under this umbrella term, Alzheimer's disease (AD) and Parkinson's disease (PD) are the most common neurodegenerative diseases that affect a large population around the globe. Alzheimer's disease is characterized by the abnormal accumulation of extracellular amyloid-*β* plaques and intraneuronal neurofibrillary tangles in brain regions and manifests as a type of dementia in aged individuals that results in memory loss, multiple cognitive abnormalities, and intellectual disabilities that interfere with quality of life. Since the discovery of AD, a wealth of new information has emerged that delineates the causes, mechanisms of disease, and potential therapeutic agents, but an effective remedy to cure the diseases has not been identified yet. This could be because of the complexity of the disease process, as it involves various contributing factors that include environmental factors and genetic predispositions. This review summarizes the current understanding on neurodegenerative mechanisms that lead to the emergence of the pathology of AD.

## 1. Introduction

Alzheimer's disease is characterized by the pathological accumulation of amyloid-*β* (A*β*) peptides and tau protein, leading to the formation of neurofibrillary tangles and the loss of neuronal cells. Amyloid beta, a 39–43-amino acid residue peptide, is found in the healthy human brain and is considered to have a normal physiological role. It exists as A*β* fibrils because of cleavage from a larger amyloid precursor protein [1]. In AD, the accumulation of amyloid fibrils as amyloid plaques or senile plaques in extracellular region of brain cells is closely linked to the loss of synapses, synaptic dysfunction, inflammatory responses, and neuronal loss. On the other hand, tau protein or T proteins are found abundantly in definite spatial patterns in the entorhinal cortex followed by hippocampal and cortical areas of the central nervous system and plays a vital role in stabilizing microtubule. In AD pathology, the tau protein undergoes intense hyperphosphorylation, leading to the clumping of tau protein, which forms intracellular neurofibrillary tangles (NFT). The intracellular formation of NFT leads to microtubule disassembly, dendritic spinal collapse, and the degeneration of axons. The pathological aggregation of intracellular NFT and extracellular senile plaques precede the initial signs of cognitive impairment in mild AD patients by at least 10–20 years. Mild signs of AD include memory loss, mood changes, nervousness, difficulty in handling money, and poor judgement. However, in late-stage AD, patients will need full time, around the clock assistance as more severe cognitive alterations, such as a loss in the ability to respond to the environment, confusion, restlessness, and difficulty with language and thoughts, as well as difficulty in the control of movement, will be experienced by patients [2].

## 2. Common Neurodegenerative Mechanisms in AD

### 2.1. Mitochondrial Dysfunction and ROS Production

Neurons in the human brain have tremendous oxygen consumption and metabolic rates. For this reason, neurons rely on mitochondria that are found in abundance in brain tissues for the production of energy by oxidative phosphorylation. Mitochondria are the central sites of ROS, as natural byproducts of the oxidative phosphorylation cascade, and excessive production of ROS is usually offset by the normal homeostasis function of mitochondria.

A*β* appears as a solitary molecule but tends to form small clusters that are soluble and able to travel freely in the brain and eventually forms plaques that are hallmarks of AD. Recent studies have highlighted the effect of A*β* on mitochondrial respiratory function, including mitochondrial complexes I–IV activities and mitochondrial oxygen consumption [3]. Chen and Yan have shown that A*β* can penetrate the mitochondrial membrane and interact with internal mitochondrial protein abnormalities and decrease the activity of the mitochondrial electron transfer chain, the citric acid cycle, and the generation of ROS [4]. Moreover, Cha et al. have demonstrated that exposure of the hippocampal cell line of mice (HT22 cells) to exogenous A*β*_1–42_ induced identical morphological alterations as those observed in A*β*PP/PS1 double transgenic mice. A*β*_1–42_ accumulation in mitochondria induces cellular toxicity and leads to cellular death [5]. Mitochondrial associated amyloid precursor protein (APP) forms a complex with the translocase of the outer mitochondrial membrane 40 (TOM40) import channel and the translocase of the inner mitochondrial membrane 23 (TIM23) import channel and causes the inhibition of nuclear-encoded cytochrome c oxidase subunits IV and Vb proteins. Following this event, cytochrome c oxidase activity is decreased and the generation of hydrogen peroxide is increased in mitochondria. This finding correlated with a higher level of regional dissemination of mitochondrial APP in AD-vulnerable areas, including the frontal cortex, hippocampus, and amygdala [6]. Studies have also shown that mitochondrial accumulated A*β* induced mitochondrial damage and neuronal death. Proteomic studies that used triple transgenic mice (pR5/APP/PS2) that carried both plaques and tangles demonstrated an enormous downregulation of proteins related to complexes I and IV of the oxidative phosphorylation system [7]. Emerging evidence supports that an interaction between the amino terminal (NH(2))-derived tau fragment of the human tau40 isoform and APP is closely associated with mitochondrial adenine nucleotide translocator-1 (ANT-1) and cyclophilin D. The two peptides inhibit the ANT-1 dependent adenosine diphosphate adenosine triphosphate (ADP/ATP) exchange and exacerbate mitochondrial dysfunction and synaptic deterioration in AD pathology [8]. However, the mechanisms related to mitochondrial impairment that lead to AD remain unclear.

### 2.2. Protein Oxidation

There is much accumulated evidence that ROS may be central to the pathogenesis of neurodegeneration in AD. The incessant build-up of ROS and RNS (reactive nitrogen species) leads to protein oxidation and lipid peroxidation. Furthermore, the human brain is composed of high levels of unsaturated lipid content and exhibits high oxygen utilization, an increased level of metal ions, and a comparatively poor antioxidant system, which makes the brain extremely vulnerable to protein oxidation and lipid peroxidation.

Protein oxidation in cell is reflected by the increased levels of protein carbonyls and 3-nitrotyrosine (3-NT). Protein carbonyls can be generated by the reaction of the superoxide anion, which leads to the fragmentation of the protein backbone. In addition, protein carbonyls may also be generated by hydrogen atom abstraction and the specific attack of ROS on several amino acid side chains, such as lysine, arginine, and proline. Studies have also demonstrated that the ROS attack on protein structure may result in the formation of Michael adducts between lysine, histidine, and cystine residues and the glycation/glycoxidation of lysine amino groups, which forms advance glycation end products (AGEs) [9, 10]. However, protein carbonyls are considered a disease marker but the source of formation by either direct protein oxidation or the addition of previously oxidised molecules cannot be differentiated. As protein carbonylation and modification cannot be repaired, these altered compounds must be eliminated from the intracellular compartments by selective proteolysis. Bota et al. have reported that the improper clearance of modified proteins and aggregates may contribute to a range of human pathologies [11]. Substantial evidence demonstrated that the protein carbonyl level was increased by 42% and 37% in the AD hippocampus and inferior parietal lobule, respectively [12]. More recently, researchers have identified other modified proteins, such as *α*-enolase, ubiquitin carboxyl terminal hydrolase L-1 (UCHL-1), dihydropyrimidinase-related protein 2, heat-shock cognate 71, creatine kinase BB, and peptidyl prolyl-*cis,trans* isomerase 1 (Pin1), in different brain regions of AD patients by using a redox proteomic approach. Importantly, recent lines of evidence have indicated that some biochemical alterations exist in proteins that play a crucial role directly or indirectly in mitochondrial energy metabolism, such as creatine kinase, triosephosphate isomerase, glyceraldehyde 3-phosphate dehydrogenase, phosphoglycerate mutase 1, and *α*-ATPase. The oxidation of these vital enzymes caused a decrease in ATP generation to maintain the normal metabolism and functions of neuronal cells, which resulted in the perturbation of ion pump and potential gradients. In addition, ATP deprivation in brain cells can cause an abnormal phosphorylation of tau protein, which can progress to AD disease onset [13].

### 2.3. Lipid Peroxidation

Lipid peroxidation refers to oxidative degradation of lipid molecules. It is a chain reaction whereby an H atom is abstracted from lipids in cell membranes by free radical species, resulting in cell membrane damage. The brain is considered vulnerable to lipid peroxidation due to its high oxygen consumption, high level of redox metal ions, reduced antioxidant defence mechanism, and high level of polyunsaturated fatty acids (PUFAs). The toxic reaction between free radicals and phospholipid-bound arachidonic acid in cell membrane produces free 4-hydroxy-2-trans-nonenal (HNE), acrolein, neuroprostanes, and isoprostanes [14]. The aldehydic product of lipid peroxidation HNE is highly toxic and can form covalent bonds with proteins through Michael adduction to amino acid cysteines, lysines, and histidines. A recent study reported elevated levels of HNE-histidine Michael adducts in the AD hippocampus and the covalent modification of the histidine side chain of A*β* results in an increased aggregation of this tau protein [15]. The aggregation of A*β* in mitochondrial membranes causes alterations and increased permeability in the membrane, which leads to the leakage of cytochrome c and the functional abnormality of the electron transport chain, which culminates in cellular apoptosis. Moreover, Tamagno et al. demonstrated in an in vivo study that increased lipid peroxidation results in increased A*β* production via the BACE 1 expression pathway [16]. Importantly, lipid peroxidation and A*β* generation in neuronal cells are capable of inducing JNK pathways, leading to programmed neuronal death. As an aldehydic product of lipid peroxidation, HNE is a highly lethal compound that impairs the coupling of metabotropic glutamate and muscarinic cholinergic receptors to phospholipase C-coupled GTP-linked proteins in cultured rat cerebrocortical neurons [17]. In addition, HNE hinders glucose transport in cultured hippocampal neurons and inhibits glutamate transport in cortical astrocytes [18]. The lipid peroxidation of arachidonic acid forms prostaglandin-like compounds known as isoprostanes. Montine et al. indicated that isoprostane levels are selectively elevated in brain tissues from patients with advanced AD, which also exhibit the deposition of A*β*, neurofibrillary tangle formation, and neurodegeneration [19].

### 2.4. Nitrosative Stress

Nitrosative stress is a condition whereby RNS generation is not counterbalanced by an array of defence mechanisms and causes damage to the intracellular components of cells. Nitric oxide (NO), the major contributor of RNS, functions as signalling molecule in regulating neural development, neurotransmitter release, and synaptic plasticity [20]. NO is produced by three types of nitric oxide synthase enzymes (NOS), namely, inducible nitric oxide synthase (iNOS), endothelial NOS (eNOS), and neuronal NOS (nNOS). RNS arise from a combination of the superoxide radical (^•^O^−^) and NO that forms a nitric oxide radical (^•^NO^−^), a peroxynitrite anion (ONOO^−^), nitrogen dioxide (NO_2_^−^), and Dinitrogen trioxide (N_2_O_3_) [21].

Nitrosative stress has been implicated in marked cognitive impairment that is associated with synaptic dysfunction and glial activation. A substantial body of evidence has indicated that A*β*_1–40_ in an AD mice model promoted the activation of iNOS and tumour necrosis factor-*α* (TNF-*α*) that subsequently led to the activation of c-Jun-NH2-terminal kinase (JNK) and Nuclear Factor-KB and significant learning and memory impairment [22]. Emerging studies have suggested that the dynamics of mitochondria fission and fusion are essential in maintaining mitochondrial function as well as maximizing bioenergetic capacity. However, nitric oxide produced in response to A*β* in AD has been found to induce mitochondrial fission thorough the S-nitrosylation of dynamin-related protein 1 (SNO-Drp1), causing synaptic impairment and neuronal damage [23]. Moreover, A*β* induced dendritic spine loss in AD has been found to be mediated by the activation of Cdk5 by the S-nitrosylation of thiol groups of cysteine residues to form SNO-Cdk5. Increased SNO-Cdk5 activity has been linked to the pathogenesis of AD, as increased levels of SNO-Cdk5 were detected in postmortem AD brain samples but not in normal samples [24]. Along this line, numerous studies have been carried out to identify the different types of protein S-nitrosylation in the progression of AD. Zahid et al. have demonstrated that 45 endogenous nitrocysteines primarily involved in neuronal metabolism, signalling pathways, apoptosis, and redox regulation were identified from the hippocampus, substantia nigra, and cortex of AD patients. Furthermore, extensive neuronal atrophy in the AD brain with increased protein S-nitrosylation and a significant number of cysteine modification sites facilitates the understanding of the involvement of S-nitrosylation in nitrosative stress induced AD pathology [24, 25].

### 2.5. Protein Aggregation

AD is the most prevalent neurodegenerative disease that is characterized by protein aggregation and inclusion body formation. Alzheimer's disease is identified by two main pathological lesions: the deposition of neuritic plaques that consist of amyloid-*β* and intraneuronal neurofibrillary tangles that consist of hyperphosphorylated tau protein.

### 2.6. Amyloidopathy

The deposition of amyloid beta (A*β*) peptide, the main component of senile plaques in neuronal cells, is known as “amyloidopathy.” It is becoming clear that the extracellular aggregation of A*β* plaques is closely associated with widespread neuronal atrophy and the concomitant damage of synapses in different brain regions that results in gradual neuronal death and memory loss. An initial study by Hardy et al. has documented that several environmental factors, including oxidative stress, drug and brain injury, and genetic variants, can contribute to the pathogenesis of AD.

The amyloid cascade hypothesis, originally presented by Hardy and Higgins in 1992, is the most accepted and well understood mechanism of deposition of the amyloid beta protein in Alzheimer's pathology. Amyloid precursor protein (APP) is a large transmembrane protein that produces A*β* that consists of either 40 or 42 amino acids. A*β*_40_ is a commonly found protein molecule whereby A*β*_42_ is highly toxic to neuronal cells. In physiological states, the membrane glycoprotein APP plays a crucial role in synapse formation and neuronal activity and transmission via two proteolytic pathways: “nonamyloidogenic” and “amyloidogenic” pathways [26]. In the “nonamyloidogenic” pathway, soluble APP*α* is released to the extracellular compartment through APP cleavage that involves *α*- and *γ*-secretases. On the other hand, the “amyloidogenic” pathway is triggered by *β*-secretase 1 (*β*-site amyloid precursor protein cleaving enzyme 1, BACE-1) mediated cleavage that produces soluble extracellular APP*β*, which is subsequently cleaved by a *γ*-secretase complex that releases A*β* into the extracellular space [27]. Subsequently, A*β* is transported and degraded by the ApoE2 and ApoE3 isoform and cleared completely through the blood brain barrier together with insulin degrading enzyme (IDE) or with the neprilysin degradation pathway (NDP). However, the binding of A*β* to the ApoE4 isoform leads largely to A*β* aggregation in the extracellular region of the central nervous system. In normal conditions, during A*β* generation, there is a feedback mechanism that releases the APP intracellular C-terminal domain and increases the level of neprilysin, which promotes A*β* turnover. Perturbation of the physiological pathway may occur in the event of mutations and changes in the expression of APP, BACE-1, IDE, Apo-E, and neprilysin lead to the progressive accumulation of A*β* and the manifestation of AD [28].

### 2.7. Tauopathies

Tauopathies are diseases associated with the intracellular pathological aggregation of tau protein in neurofibrillary tangles, which is observed in almost 26 neurodegenerative diseases, including Alzheimer's disease, progressive supranuclear palsy (PSP), Dementia Pugilistica, and Pick's disease. In AD, the paired helical filament type of tau aggregates and forms tangles in neuronal cells, which leads to neurodegeneration and leaves the tau aggregates appearing as neurotoxic “ghost-tangles” [29]. It has been widely understood that tau is a microtubule binding protein that is found largely in neurons, specifically in axons and dendrites. Tau protein functions in the maintenance of microtubule stabilization, cellular morphology, and the axonal movement of organelles and vesicles [29]. The expression of six isoforms of the tau protein in human neurons is regulated by the microtubule-associated protein tau (MAPT) gene in chromosome 17. The six isoforms are 4R2N, 4R1N, 3R2N, 4R0N, 3R1N, and 3R0N, and they are produced as a result of alternative mRNA splicing of the MAPT gene; the shortest form, the 3R0N isoform, is expressed predominantly in the foetal brain. These 6 isoforms differ from one another by the inclusion and exclusion of one or two inserts (29 or 58 amino acids) in N-terminal sequences and by the presence or absence of a long microtubule binding repeat domain in the C-terminal half [30]. The loss of functional tau due to hyperphosphorylation state of paired helical filament (PHF) of all 6 isoforms of tau proteins is the pathological hallmark of AD which leads to disruption in microtubule assembly, decreased axonal transport, and neurodegeneration in areas such as frontotemporal lobe of human brain. Köpke et al. have demonstrated that cytosolic hyperphosphorylated tau from AD patients contains 5 to 9 mols of phosphate per mol of tau protein in comparison to normal tau, which only contains two to three mols of phosphate per mol of tau protein [31].

Emerging studies have found a correlation between a decrease in brain insulin signalling and the level of hyperphosphorylation of tau protein in AD. These data were further bolstered by epidemiological studies that showed that diabetic patients with a high level of glycated proteins have a higher risk of developing AD. Hence, the increased glycosylation of tau protein results in tau protein that is more susceptible to phosphorylation by cAMP-dependent protein kinase (PKA), glycogen synthase kinase-3*β* (GSK-3*β*), and cyclin-dependent protein kinase 5 (cdk5), but is also more sensitive to the downregulation of dephosphorylation by protein phosphatase 2A (PP-2A). The activity of PP-2A is markedly reduced in AD, which could be one of the contributing factors of the abnormal hyperphosphorylation of the tau protein. These toxic proteins cause the inhibition and breakdown of microtubule structures in neurons and further cause impairment in axonal transport, leading to a “domino” cascade of events that result in dementia [32].

A plethora of scientific evidence has clearly indicated that an increased generation of reactive oxygen species (ROS) that is not counterbalanced by an internal defence mechanism results in oxidative stress and the manifestation of neurodegeneration diseases. Interestingly, recent studies have demonstrated a crosslink between mitochondrial oxidative stress and the hyperphosphorylation of tau protein. Studies carried out on null mice that lack the superoxide dismutase 2 (SOD2) gene showed that treatment with a catalytic low dose of antioxidant resulted in the elevation of tau phosphorylation specifically at the Ser-396 amino acid sequence. However, tau phosphorylation was halted with the administration of a high dose of antioxidant supplement, which indicates that an efficient internal antioxidant defence system prevents tau hyperphosphorylation and the manifestation of AD neuropathology [33]. These* in vivo* findings are also supported by* in vitro* studies whereby phosphorylated tau was markedly increased via the inhibition glutathione synthesis in M17 neuroblastoma cells [34].

### 2.8. DNA Damage

Deoxyribonucleic acid (DNA) is located in the nucleus and mitochondria of living cells. The maternally inherited human mitochondrial DNA (mtDNA) is circular in shape with a size of approximately 16.5 kb and encodes 13 polypeptides, 22 tRNAs, and 2 rRNAs. These proteins play crucial roles in orchestrating the oxidative phosphorylation pathway through the electron transport chain to generate ATP. Several lines of evidence have suggested that nuclear and mitochondrial DNA are subjected to genotoxic assault by ROS produced as a byproduct of the oxidative phosphorylation cascade and exposure to environmental toxins and radiation. As mtDNA is located in close proximity to the oxidative phosphorylation cascade, studies have suggested that mtDNA is more susceptible to genotoxic assault than nDNA. However, more studies should be carried out to understand the actual reason for the increased susceptibility of mitochondrial DNA damage in comparison to nDNA. Therefore, to overcome DNA damage, mammalian mitochondria are equipped with inimitable DNA repair pathways that respond to oxidative DNA damage. Whether these repair proteins are of nuclear origin or preexist in mitochondria at the point of oxidative DNA damage is not clearly understood. The damaged DNA is subjected to DNA repair pathways that include base excision repair (BER), direct reversal, mismatch repair, translesion synthesis, and double-strand break repair [35].

Tau protein plays a key role in safeguarding the integrity of neuronal genomic DNA from ROS and oxidative stress in addition to its role in microtubule dynamics. An alteration in tau protein may result in a loss of nucleic acid safeguarding functions and increase the susceptibility to ROS induced oxidative damage in genomic DNA and nuclear RNA in hippocampal neurons in AD patients. ROS have been shown to cause DNA strand breaks and to contribute to downstream events in the disease mechanism of AD. This correlation was suggested by Mullaart and colleagues; they demonstrated a 2-fold increase in DNA damage in AD brain neurons and suggested that this could be the earliest detectable pathological changes in the progression from normal to AD brain [36]. Along these lines, an increase in damaged DNA bases, namely, 8-hydroxyadenine, 8-hydroxyguanine, Fapy-guanine, 5-hydroxyuracil, and Fapy-adenine, was identified in the parietal, temporal, occipital, and superior gyrus and in the hippocampus region in patients with AD. The most protuberant DNA marker discovered in most biological samples, such as blood cells, urine, and brain specimens, is 8-hydroxyguanine (8-OHG). Mecocci and colleagues have demonstrated a significant 3-fold increase in the level of the DNA marker 8-OHG in the parietal cortex region in AD patients compared to normal individuals, specifically in mitochondrial DNA (mtDNA) compared to nuclear DNA [37]. Essentially, in normal cells the 8-OHG oxidative adduct is recognized and excised by a base repair protein, 8-oxoguanine-DNA glycosylase (OGG1) [38]. OGG1 is a bifunctional enzyme with both DNA glycosylase and apurinic/apyrimidinic lyase activities. A substantial body of evidence has identified two mutations of OGG1 that are single-nucleotide polymorphisms as a result of amino acid substitutions A53T and A288V. In 2007, these A53T and A288V polymorphic OGG1 proteins were detected in brain tissue from late-stage AD patients but were absent in a control group [39]. Tissues with A53T polymorphic OGG1 that were found to have decreased catalytic activity due to a reduced ability to bind DNA substrates and A288V protein demonstrated decreased lyase activity, leaving the DNA more susceptible to oxidative damage [40].

### 2.9. Depletion of Endogenous Antioxidant Enzymes

**Figure 1:**
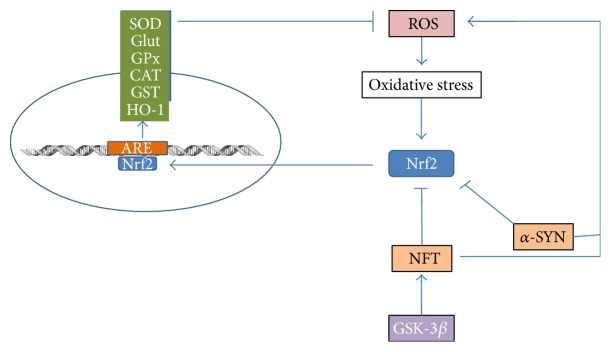
Activated Nuclear Factor 2 (Nrf2) by oxidative stress enters nucleus and escalates the transcription of ARE-containing genes. These genes cause the expression of antioxidant enzymes, that is, SOD (superoxide dismutase), Glut (glutathione), GPx (glutathione peroxidase), CAT (catalase), and GST (reduced glutathione). Subsequently, these enzymes will catalyse the detoxification of ROS and protect neuronal cells from damage. Glycogen synthase kinase-3*β* participates in phosphorylation of tau protein and induce accumulation of neurofibrillary tangles (NFT) that inhibits Nrf2. Besides inhibiting NRf2 activity, alpha-synuclein and NFT stimulate oxidative stress via ROS production.

Aerobic cells are inherently equipped with natural enzymatic and nonenzymatic antioxidant systems that prevent the accumulation of ROS. The enzymatic antioxidant system is a vital component that comprises enzymes such as superoxide dismutase (SOD), glutathione peroxidase, and catalase, which catalyses the ROS to less toxic molecules and thereby plays a key role in preventing lipid peroxidation. The superoxide anions, which are produced during normal cell signalling processes, are converted to hydrogen peroxide (H_2_O_2_) and oxygen molecules (O_2_) by SOD. On the other hand, glutathione peroxidase functions in detoxifying hydroperoxides, including DNA and lipid hydroperoxides, into water and alcohol molecules. This enzyme catalyses the reduction of hydroperoxides at the expense of reduced glutathione (GSH) to produce oxidised glutathione (GSSG) and reduced hydroperoxide. Subsequently, the GSSG is recycled to GSH by glutathione reductase and NADPH. In addition, catalase detoxifies H_2_O_2_ molecules, which are converted to oxygen and water molecules. Oxidative stress in cells activates the transcription of antioxidant enzymes through the antioxidant response element (ARE) and the binding of members of the Cap-N-Collar family of transcription factors, including Nuclear Factors Nrf1 and Nrf2 [41]. The Nuclear Factor erythrocyte derived-2 (Nrf2) plays a crucial role in the activation of over 250 genes with a common cis-acting enhancer known as ARE. These genes function in detoxification reactions that include glutathione and peroxiredoxin/thioredoxin metabolism, the generation of NADPH via the pentose phosphate pathway, proteostasis, and lipid peroxidation. Under physiologic conditions, the level of Nrf2 is low in cells due to its constant turnover. However, during oxidative stress caused by different stimuli, the level of Nrf2 increases and it enters the nucleus to escalate the transcription of ARE-containing genes [42]. A substantial amount of evidence has suggested that several proteinopathies, including tauopathies and synucleopathies, are associated with a reduced level of Nrf2 activity. In AD, TAU kinase (GSK-3*β*) participates in the phosphorylation of tau protein in microtubules, which results in the accumulation, aggregation, and formation of neurofibrillary tangles that induce an abnormal production of free radicals or ROS (Figure 1). Indeed, the increased activity of GSK-3*β* impairs the appropriate response of Nrf2, which in turn inhibits the activation of ARE associated gene activation and reduces the level of antioxidant enzymes in affected cells. Moreover, studies have also shown that the level of Nfr2 is extraordinarily high in the cytoplasm of neurons located in the hippocampus region in patients with AD, leading to the reduced transcriptional activity of Nrf2 and a lack of ARE gene activation. Nevertheless, compelling data from recent studies suggested that a reduction in SOD exacerbates neuronal and vascular pathology via the development of cerebrovascular amyloidosis, gliosis, plaque-independent neuritic dystrophy, and increased transcription factor Nuclear Factor kappaB activity in human amyloid precursor protein (hAPP) transgenic mice [43]. In addition, studies have shown that catalase-A*β* peptide interaction and colocalization decreased the efficiency of catalase in offsetting cellular damage induced by the ROS intermediate H_2_O_2_ in a human neuroblastoma cell line [44]. However, there is no significant alteration in glutathione peroxidase enzyme activity in autopsied brain regions of AD patients, although increased glutathione peroxidase activity was detected in erythrocytes [45].

### 2.10. Proteosomal Dysfunction

**Figure 2:**
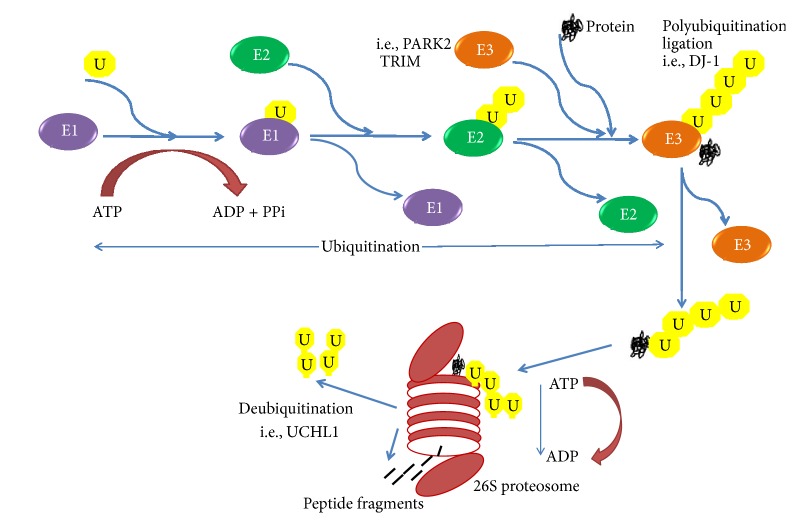
Protein degradation by ubiquitin-proteasome pathway. E1, E2, and E3-ubiquitinating enzymes.

The ubiquitin-proteasome pathway (UPP) functions in maintaining cellular integrity through the removal of abnormally folded or aggregated proteins. The disruption in this system in clearing unwanted protein aggregates results in accumulations of toxic and misfolded proteins in neuronal cells, which is regarded as pathological hallmark of AD. The survival and longevity of neurons are determined by the effective removal of abnormal protein trash by this pathway. The UPP is comprised of ubiquitin, the 26S proteasome, ubiquitin activating enzyme (E1), ubiquitin conjugating enzyme (E2), ubiquitin ligating enzyme (E3), and deubiquitinating enzymes (DUB). Ubiquitin, an 8.5 kD protein 76-amino acid chain found in all eukaryotic cells, acts as a covalent tag by marking the misfolded or short-lived proteins to be degraded by UPP. Initially, ubiquitin is activated and transferred by E1 to E2, which permits the conjugation of the target protein with ubiquitin. This reaction is mediated by E3 ligase enzymes; ubiquitin molecules are connected to the initial ubiquitin, generating polyubiquitin chains. The polyubiquitin adducts serve as a preferred substrate for proteolysis by 26S proteasome [46]. Finally, ubiquitin protein is recycled and reused to carry more damaged proteins by deubiquitinating enzymes, including ubiquitin C-terminal hydrolases (UCH-L1, UCH-L3, and UCH-L5) and ubiquitin-specific proteases (USP7 and USP14). Once deubiquitinated, the damaged protein will enter the 26S proteasome and undergo proteolysis to amino acids by the 20S core particle. Therefore, the normal function of UPP is highly crucial for the consistent clearance of abnormal protein from the neuron to prevent the deposition of abnormal proteins that trigger the onset of neurodegenerative diseases [47] (Figure 2).

Recent studies have shown that the intracellular deposition of phosphorylated tau and A*β* protein aggregates causes a direct impairment of UPP in AD patients. Proteosome activity is significantly decreased in brain tissue collected from early AD patients. The intraneural deposition of paired helical filaments in AD causes inhibitory binding of this protein on the proteasome and results in a 56% decrease in proteasome activity in the gyrus of AD patients. Hence, the inability of UPP to clear the phosphorylated tau and paired helical filament directly induces neuronal damage in the AD brain [48]. In addition, the activities of E1 and E2 in the cytosol of AD patients were almost undetected in postmortem samples of cerebral cortex from AD patients compared to normal controls. These data were further substantiated with an adult rat fraction, in which enriched ubiquitin enzymes were found to restore the capacity of the AD brain cytosolic fraction to produce conjugates. Lam et al. reported that a mutant form of ubiquitin has been observed in the brains of AD patients but not in a normal control group. The mutant ubiquitin is a substrate for polyubiquitination but is resistant to disassembly by deubiquitinating enzymes, which inhibit degradation by 26S proteasomes, resulting in the intracellular build-up of mutant ubiquitin, leading to AD pathology [49]. Consistent with this data, recent studies have demonstrated the close association between hyperphosphorylation and ubiquitinated synaptic tau, which forms stable oligomers with increased proteasome components, suggesting a dysfunction in ubiquitin-proteasome system in AD [50]. Furthermore, emerging studies have shown that a substantial decline in proteasome activity was detected in autopsy brain samples of AD patients, particularly in the hippocampus and parahippocampal gyrus regions, but not in the occipital lobe or cerebellum regions. The difference in proteasome activity in AD brains is attributed to the inhibition of proteasome activity in certain brain regions as a result of posttranslational modification [51]. Although large number of studies have shown the close association between accumulation of hyperphosphorylated tau and dysfunctional UPP, none of the studies have pointed out whether it is the hyperphosphorylated tau that causes impairment of UPP machinery or vice versa. Hence, more studies using cellular models must be designed to identify the trigger point of pathological event that results in abnormal neuron activity in AD patients.

### 2.11. Microglial Activation

Glial cells are nonneuronal cells that form the microarchitecture of the brain matter and work closely with their active partners, astrocytes. Microglia are a type of glial cells that account for 10–15% of all cells present in brain. Essentially, microglia are widely accepted as innate immune cells of the central nervous system and play a significant role in defending the brain from pathological insult. Microglia appear in a “resting state” in the normal or healthy brain and under different stimuli, they are activated to a classical (M1) or alternative (M2) state. The activated M1 state microglia cells secrete proinflammatory cytokines, such as tumour necrosis factor-*α* (TNF-*α*), interleukin-6 (IL-6), chemokines, ROS, interleukin-1*β* (IL-1*β*), and acute phase proteins, to eliminate the invading pathogens. On the other hand, the M2 activated microglia maintain homeostasis by downregulating the effects of proinflammatory cytokines through the secretion of anti-inflammatory cytokines. Intriguingly, activated M1 microglia cells have been demonstrated to surround amyloid plaques in AD brain tissues and triggering proinflammatory responses in an attempt to clear A*β* from the brain. Moreover, the resulting release of cytokines, complement activation, and phagocytosis exert detrimental effects on the surrounding neuronal cells by disrupting synaptic plasticity, promoting tauopathies, and suppressing the microglial clearance of A*β*, which leads to neuronal loss in AD [60].

Most recently, a genome-wide association study (GWAS) has identified a novel association of the HLA-DRB4-DRB1 region (that encodes MHC-II) with the late onset of AD [61]. In another GWAS, the CD33 gene was found to modify microglial function in AD (though not entirely) as it plays a role in immune responses [62]. However, the most striking GWAS identified a variant in TREM2 (triggering receptor expressed on myeloid cells 2) as a risk factor for the late onset of AD [63]. TREM2, together with DAP12, plays key role in mediating the phagocytosis of invading pathogens and apoptotic cells and suppresses inflammatory cytokine expression triggered by Toll-like receptor-2 (TLR-2) and TLR-4 signalling. Although both TREM2 and DAP12 are increased in late onset AD, the mechanism of how these receptors regulate microglial activation is not clear [63, 64].

### 2.12. Neuroinflammation

Neuroinflammation is a complex sequence of inflammatory events in nervous tissue that are triggered in response to infection, trauma, and toxic substances. In the past 20 years, a wealth of information has emerged associating aging and AD with neuroinflammation (Table 1). In addition to neuronal cells, microglia and astrocytes are major cells that are involved in inflammatory events in the central nervous system. The initial discovery was made by McGeer et al., by demonstrating the presence of reactive microglia in substantia nigral region of postmortem brain tissue of PD patients. As a consequence of persistent microglial activation that surrounds both A*β* and tau tangles, the glial cells lose their homeostatic function and attain a proinflammatory characteristic and intensify neuronal damage. In the event of neuroinflammation, inflammatory mediators, such as TNF-*α*, IL-6, IL-1*β*, and cyclooxygenase-2 (COX-2), are found in serum and brain samples of AD patients [65].

Inducible COX-2 has been found to be stimulated in rat model neurodegenerative studies. In addition, the upregulation of COX-2 gene expression was detected in the frontal cortex of AD patients. Inducible COX-2 synthesizes different types of prostaglandins (PGs) (PGE_2_, PGD_2_, and PGF2*α*) from arachidonic acid and studies have shown that PGs stimulate microglia via PG receptors that are found in microglia and trigger the production of proinflammatory cytokines. PGE_2_ is a crucial proinflammatory marker of the neuroinflammation process, as the PGE_2_ level was upregulated in the CSF of AD patients [66]. Caspases are cysteine aspartic proteases that play a significant role in neuroinflammation and cellular apoptosis. Data in support of this idea include studies by Burguillos et al. that showed that microglia can release various potentially neurotoxic proinflammatory mediators through a protein kinase C- (PKC-) *δ*-dependent pathway. These caspases were detected in activated microglia collected from the ventral mesencephalon of PD patients and from frontal cortex areas of patients with AD [55]. In light of the finding that chronic inflammation is an important histopathologic feature in AD, recent studies have revealed that the stimulation of human primary astrocytes or the astrocytoma cell line U373 with IFN*γ* and TNF*α* or IFN*γ* and IL-1*β* triggered the production of A*β*1–40 and A*β*1–42. The combination of IFN*γ* with either TNF*α* or IL-1*β* stimulated A*β* production mediated by *β*-secretase cleavage of the immature amyloid precursor protein (APP) molecule [67, 68]. On the other hand, Holmes et al. have demonstrated that an increase in serum TNF-*α* is clearly correlated with the rate of cognitive decline in AD patients compared to the low TNF-*α* level in the control group that showed no cognitive decline over the same period of time. Hence, this study further supported the hypothesis that acute episodes of systemic inflammation, particularly, TNF-*α*, are linked to long-term cognitive decline in AD [68].

### 2.13. Neuroepigenetic Modification

The term “epigenesis” was first proposed by Waddington as heritable changes in gene expression that was not originated from physical changes in DNA sequence. Hence, neuroepigenetic alterations can be explained as changes in neuronal DNA that switches “on” or “off” certain genes which contributes to the onset of pathological changes in neurodegenerative diseases, such as Alzheimer's disease. The two well established epigenetic modifications that orchestrate changes in large number of genes across multiple pathways are DNA methylation and histone modification.

DNA methylation is an extremely conserved mechanism that occurs with the transfer of a methyl group to a single stranded DNA, particularly at the 5′-C-p-G-3′ (CpG) sites. In mammalian cells, the cytosine methylation (5mC) is regulated by four types of DNA methyltransferase (DNMT) enzymes, namely, DNMT1, DNMT2, DNMT3A, and DNMT3B. The CpG methylation catalysed by DNMTs interrupts the attachment of transcription factors and allows the binding of methyl-CpG-binding domain proteins (MBDs) that are directly associated with chromatin compaction and gene silencing. A recent DNA methylation study revealed that A*β* treated DNA samples collected from neurons showed a significant change in DNA methylation load besides upregulating the apoptotic and neuronal differentiation genes. In light of this finding, it is very clear that neuroepigenetic modifications are triggered by upstream mechanism of intracellular accumulation of A*β* that leads to downstream changes in DNA methylation process that regulates changes in gene expression that exacerbate the disease process in AD [69]. Evidence in support of this comes from Chouliaras et al. that demonstrated that the levels of DNA methylation and DNA hydroxymethylation (5hmc) were significantly decreased and inversely correlated with presence of amyloid plaque load in the hippocampal subregion of AD patients. Lately, DNA hydroxymethylation has been described in the field of epigenetics as oxidation of methylated cytosine by an enzyme known as ten-eleven translocation (TET). Whereas DNA methylation is associated with silencing of gene, DNA hydroxymethylation results in increased gene expression [57]. On the contrary, significant increase in global DNA methylation was reported from DNA sample isolated from peripheral blood mononuclear cells of late onset of Alzheimer's disease (LOAD) patients. Interestingly, DNA hypermethylation levels were associated with inheritance of APOE polymorphic ɛ4 carriers, the important genetic determinants of AD risk in global population [58]. Besides that, evidence from gene sequencing studies has shown that decreased level of* triggering receptor expressed on myeloid cells 2* (TREM2) enhanced the A*β* deposition and neuronal loss in mouse model of AD. TREM2 is an important receptor expressed on microglia that functions in regulating proinflammatory response via promoting phagocytosis, degradation, and removal of toxic protein A*β* accumulation in AD brains. Mutation or aberrations that give rise to single-nucleotide polymorphism (rs75932628) within TREM2 are associated with disruption in the expression of TREM2 receptor that triggers inflammatory response, oxidative radicals, and neurotoxin production and eventually production of A*β* protein [59]. According to Lue et al., TREM2 protein expression was remarkably correlated with increased phosphorylated tau and proinflammatory mediator, caspase 3 in postmortem temporal cortical samples of AD patients [70]. Consistent with this finding, Ozaki et al. have reported that the increase in TREM2 mRNA expression was negatively correlated with DNA methylation at CpG sites in intron 1 of TREM2 region in leucocytes of AD patients compared to that in control. Therefore, DNA methylation status may contribute to alteration in gene expression that exacerbated the neuroinflammation process, phosphorylated tau formation, and apoptosis of neurons leading to AD [59]. Nevertheless, studies have shown the levels of 5mC and 5hmc were markedly increased in human middle frontal gyrus and middle temporal gyrus in AD patients alongside phosphorylated amyloid beta, tau, and ubiquitin proteins. However, the levels of 5hmc and 5mc were significantly lower in astrocytes and microglia compared to remarkable elevation in neurons. Hence, it is of the utmost importance to determine the time frame of sample collection in DNA methylation study as the level changes during advancement of AD pathology and this information can be used to develop biomarker for early diagnosis of the disease and as a therapeutic approach [71].

Histone modifications, such as histone acetylation and deacetylation, are two types of epigenetic changes that are responsible for preserving the chromatic stability. Histone acetylation is regulated by two opposing enzymes, histone acetyltransferases (HATs) and Histone deacetylases (HDACs). HATs are responsible for addition of an acetyl group to lysine residues at the N terminus of histone proteins and reveal the chromatin structure allowing easy accessibility for transcription factor binding increased gene expression. Conversely, HDACs remove acetyl groups and maintain closed chromatin structure with gene transcription repression. Overwhelming studies have suggested that free radicals and oxidative stress resulting from aging, hypoxia, and hyperglycemia may spiral out of control resulting in A*β* and NFT induced neuronal death. Recent studies have demonstrated that hydrogen peroxide activated the histone acetyltransferases p300/CAMP-response element binding protein (p300/CBP) and downregulated the HDAC3, which might indirectly activate enzyme-dependent global histone hyperacetylation. The histone hyperacetylation via CBP/p300 cascade played a major role in the upregulation of inflammatory mediator, NF-*κ*B, that mediates gene expression for A*β* production under oxidative stress [72]. Along with this, histone hyperacetylation has been found to activate stress-related gene pathways that could upregulate the transcription of* APP*,* BACE-1*, and* PS1* genes, therefore causing overproduction of A*β* in AD [73]. In accordance with this data, Narayan et al. have demonstrated that acetyl histone H3 and acetyl histone H4, including total histones H3 and H4, were remarkably increased in pyramidal neurons from postmortem AD patients. Intriguingly, a significant correlation was discovered between histone hyperacetylation and compromised protein degradation pathway resulting in build-up in ubiquitin load and cell death [74]. Similarly, Lithner et al. have revealed that histone hyperacetylation was observed in histone H3 at lysine 9 of postmortem neocortex (occipital cortex) of AD patients [75]. Surprisingly, studies conducted on animal model of AD showed significantly decreased H3 and H4 histone acetylation that was induced by accumulation of A*β* that affected reduction in neuroligin 1, a critical gene involved in synaptic plasticity causing memory deficit [76]. In addition, Gjoneska et al. have reported the decreased H3K27 acetylation at regulatory regions of synaptic plasticity genes in the p25 transgenic model of AD [77]. In order to counteract the decreased acetylation in histone, HDAC inhibitors have been used to improve histone acetylation that in turn alleviates the detrimental effect of A*β* induced neurotoxic effect in mouse model of AD. A number of HDAC inhibitors including 4-phenyl butyrate, valproic acid, trichostatin A, and suberoylanilide hydroxamic acid (SAHA) have been reported to increase histone acetylation in mouse model of AD. Fontán-Lozano et al. have reported that HDAC inhibitors reversed learning and consolidation deficit via increasing H3 histone acetylation that causes modulation of genes that are responsible for memory and learning in mice model of AD [76]. It is quite puzzling, on the role of HDAC inhibitors in elevating histone acetylation in human brain, as recent studies have shown that histone acetylation is greatly elevated in postmortem samples of AD [74]. Consistent with this idea, the effect of HDAC inhibitor, valproate, was tested on a randomized, double-blind study using AD patients, and the study reported that valproate is not effective in the management of agitation and aggression. Moreover patients experienced significant worsened adverse reaction in postvalproate therapy [78]. Therefore, more research should be aimed at elucidating the role of HDAC inhibitors in histone acetylating enzymes along with other pathways such as inflammation, oxidative stress, and microglia activation as more solid understanding will be gained on the molecular changes exerted by HDAC inhibitors. Besides that, more clinical studies should be conducted to verify the efficacy of HDAC inhibitors as a potential therapeutic strategy in mitigating synaptic microtubule damage and memory impairment in AD patients.

## 3. Conclusion

This review summarized the current concepts on neurodegenerative pathways that may lead to a “domino” cascade of events leading to manifestation of AD. Aging and oxidative stress are currently recognized as key factors that cause the pathophysiological features of these diseases. The inevitable aging process may augment ROS formation and accumulation, which activate several neurodegenerative pathways and lead to neuronal loss. Multiple pathways have been recognized to cause AD, but these changes are not seen in all cases of AD. Treatment strategies can be achieved by targeting the specific pathways that were activated, such as halting the deleterious neuroinflammatory pathway, improving the clearance of aggregated protein by UPP, and augmenting the endogenous antioxidant enzyme level.

It is extremely important to understand the intricate details of disease mechanism, as this understanding allows researchers and clinicians to identify the right therapeutic approach and disease management at an early stage; late stage of AD usually has a lower success rate of treatment. To be diagnosed at an early stage of the disease, it is essential to define the biomarkers that correctly indicate the onset of the disease in patients. Disease biomarkers can also be used to monitor the severity of the disease and the response to prescribed drugs. Efforts to discover more diagnostic biomarkers should be encouraged and integrated with state-of-the-art approaches to experimental and clinical trial designs to discover suitable treatments for these diseases. Given the explosive amount of information on AD, we are sure that the discovery of an ideal therapeutic drug for AD will be achieved in the near future.

## Figures and Tables

**Table 1:** Evidence of inflammatory mediators reported in AD.

Inflammatory mediator	Changes reported	Human site	References
Tumour necrosis factor-*α* (TNF-*α*)	↑	Serum	[52]
	↑	Brain	
	↑	Brain	
	↑	Blood	

Interleukin-6 (IL-6)	↑	Serum	[53]
	↑	Brain	[54]
	↑	Serum	[55]
	↑	CSF	

Interleukin-1*β* (IL-1*β*)	↑	Brain	[56]
	↑	Blood	[55]
	↑	CSF	

Cyclooxygenase-2 (COX-2)	↑	Neurons	[57]

Interleukin-2 (IL-2)	↑	Serum	[55]
	↑	CSF	

Monocyte chemotactic protein (MCP-1)	↑	Brain	[52]
	↑	Blood	

Caspase-8	↑	Neurons	[57]
	↑	Microglia	[58]

Caspase-3/7	↑	Microglia	[58]

Transforming growth factor (TGF) *β*1	↑	CSF	[59]

Transforming growth factor (TGF) *β*2	↑	CSF	[59]
